# Unilateral hypertensive choroidopathy as a sole manifestation in malignant hypertension: optical coherence tomography angiography findings-case report

**DOI:** 10.1186/s12886-023-02970-w

**Published:** 2023-05-22

**Authors:** Dimitrios Karagiannis, Nikolaos Bouratzis, Loukas Kontomichos, Panagiotis Pantazis, Stylianos Kandarakis, Efstratios Paroikakis

**Affiliations:** 1 Second Department of Ophthalmology, Ophthalmiatreion Eye Hospital of Athens, Athens, Greece; 2 Department of Ophthalmology, National and Kapodistrian University of Athens, 1st University Eye Clinic, G. Gennimatas General Hospital, Athens, Greece

**Keywords:** Malignant hypertension, Hypertensive choroidopathy, OCT-angiography, Exudative retinal detachment, Choriocapillaris

## Abstract

**Background:**

We present a case of hypertensive choroidopathy due to malignant hypertension with exudative retinal detachment as a sole finding. We use OCT- angiography for initial diagnosis and report findings from extensive follow up.

**Case presentation:**

A 51-year-old female with no past medical history, presented to our clinic with painless loss of vision in her left eye. Fundus examination revealed only exudative retinal detachment in her left eye that was confirmed with Optical Coherence Tomography. Fluorescein angiography showed hyperfluorescent spots with leakage in late phases. OCTA manifested a focal dark area in the choriocapillaris slab corresponding to flow signal voids, signifying regions of non-perfusion. Her blood pressure was 220/120 mmHG. Complete blood work -up failed to reveal any other possible etiology. During follow-up period of 9 months blood pressure normalized, patient regained visual function and choriocapillaris perfusion was completely restored.

**Discussions and conclusions:**

Hypertensive choroidopathy with exudative retinal detachment can be the only sign of malignant hypertension and no pre-existing history of a systemic disease is required in order to become apparent. OCTA reveals areas of non-perfusion at choriocapillaris level, proving that it is an essential tool in the diagnosis and follow up of patients with hypertensive choroidopathy. Finally, we propose that early diagnosis prevents permanent damage of the RPE and leads to complete choroidal remodeling and better visual outcomes.

## Introduction

The European Society of Cardiology defines hypertension emergencies as situations in which severe hypertension (systolic BP > 180 mmHg and/or diastolic BP > 110 mmHg) is associated with acute hypertension mediated organ damage. Malignant hypertension one of the typical presentations of hypertension emergencies is characterized by severe hypertension associated with fundoscopic changes and microangiopathy [1].

Untreated accelerated hypertension can cause retinopathy, optic neuropathy and/or choroidopathy [2, 3]. Hypertensive choroidopathy, as a distinct entity, is far less common than retinopathy/chorioretinopathy and usually appears in younger patients because of elasticity of their blood vessels [3]. Typical funduscopic signs include exudative retinal detachment, Elschnig spots and Siegrist streaks [3].

Optical Coherence Tomography Angiography (OCTA) represents a novel non-invasive imaging tool, which enables us to perceive all retinal and choroidal layers separately, in contrary to the conventional angiographies which due to their two-dimensionality lack that capacity [4]. Limited data in literature demonstrate the value of OCTA in the evaluation of hypertensive chorioretinopathies.

This is an interesting case of hypertensive choroidopathy with exudative retinal detachment as a sole finding, in a patient with no past medical history. Multimodal imaging including OCTA was used for the initial diagnosis with a follow-up period of 9 months.

## Case report

A 51-year-old Caucasian female, presented to our Emergency Department with a 5-day history of painless loss of vision in her left eye and reported no other systemic symptoms such as headache, dizziness or shortness of breath. Patient reported no other known past medical history, other than an allergic reaction to iodine. Best corrected visual acuity was 20/20 in her right eye and 20/70 in her left eye (Snellen acuity chart). Sitting blood pressure was taken from the left arm three times with an average measurement of 220/120 mmHg. Intraocular pressure was 17 mmHg and 18 mmHg in her right and left eye, respectively. Pupils were normal with no afferent pupillary defect. Slit lamp examination of the anterior segment, revealed no signs of inflammation in the anterior chamber and no anatomical abnormalities in both eyes. Fundus examination of the left eye revealed only a distinct yellowish, circular elevation of the retina associated with exudative retinal detachment. Neither disc swelling nor any other signs of hypertensive retinopathy were identified during fundoscopy. Findings in the right eye were unremarkable.

**Fig. 1:**
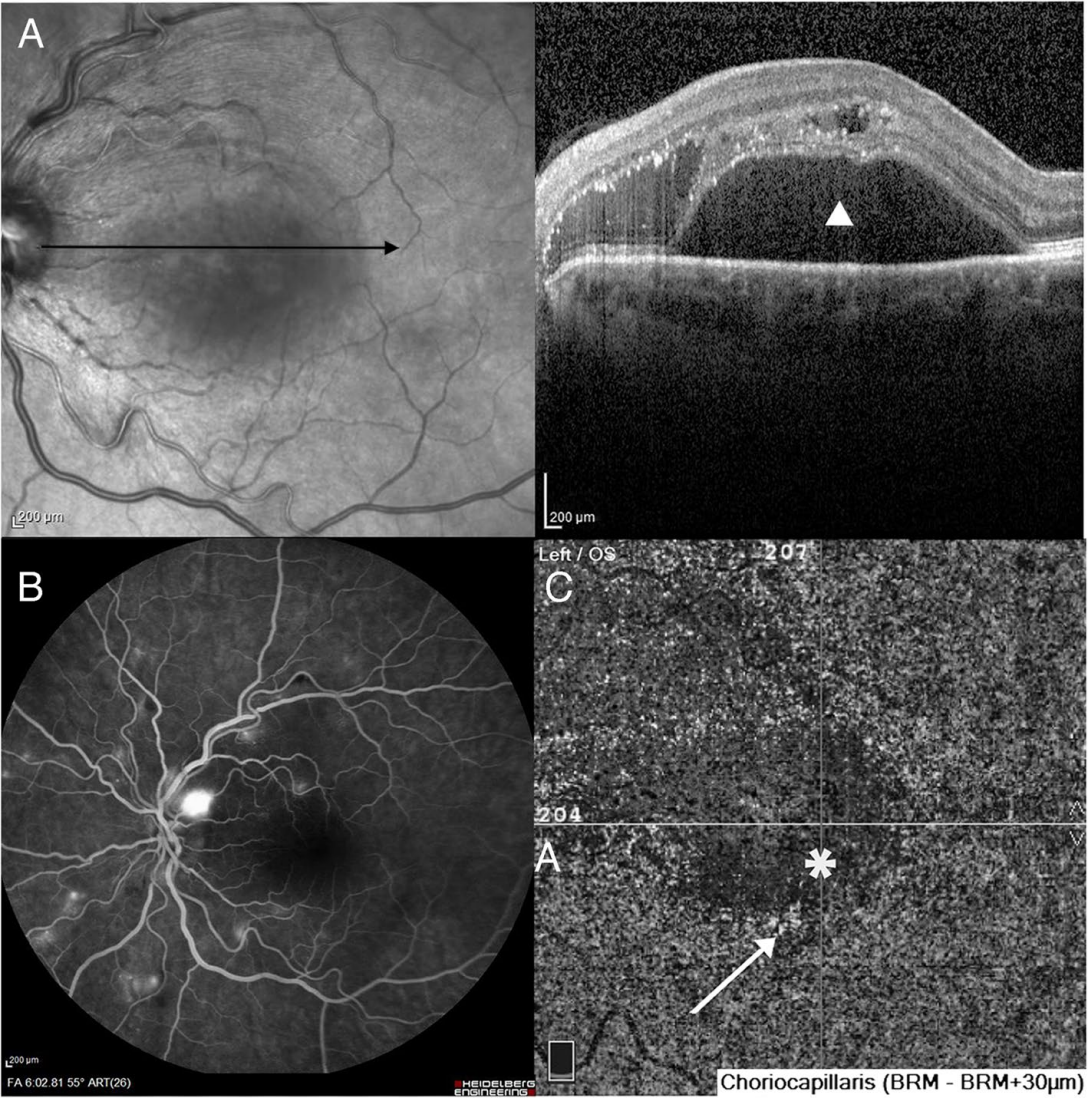
Exams at baseline. **A** SS-OCT B scan demonstrates extensive exudative retinal detachment involving the macula. (triangle) **B**) Fluorescein Angiography-Late phase (6 min) shows bilateral hyperfluorescent spots with leakage in the left eye. **C** OCTA 6..0 × 6.0 slabs demonstrate a dark area (asterisk) corresponding to flow voids in the CC slab and hyperreflective foci surrounding that area (arrow)

**Fig. 2:**
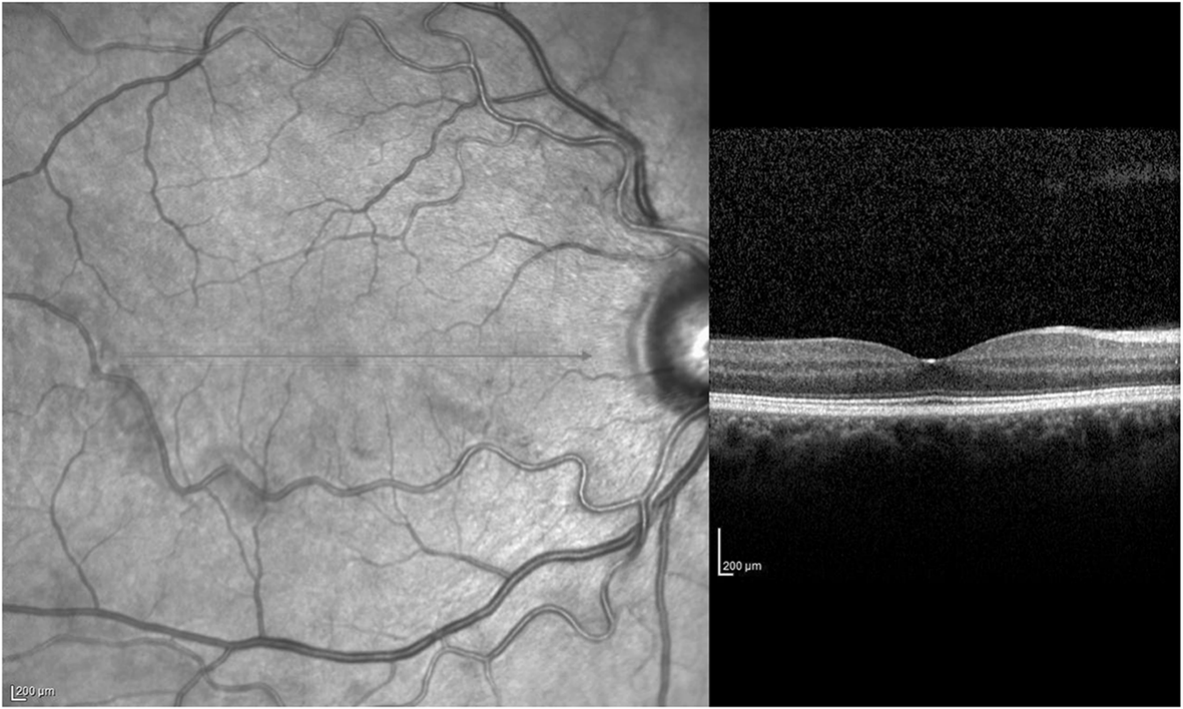
SS-OCT of the right eye. Patients right eye was normal at initial visit and during follow-up

Spectral Domain (SD) OCT B-scan through the macula displayed intra-retinal and subretinal fluid corresponding to the exudative retinal detachment (Fig. 1A) Fluorescein angiography (FA) was performed in both eyes using a Spectralis HRA2 device (Heidelberg Engineering, Heidelberg, Germany). FA showed numerous hyper-fluorescent spots demonstrating leakage in late phases of the exam (Fig. 1B). OCT B-scan and FA in the right eye were normal (Fig. 2). Indocyanine Green Angiography was not performed because of patient’s allergy to iodine.

OCTA using AngioVue™ (Optovue Inc., Fremont, CA, USA) was also performed. The Superficial and Deep slabs in OCTA 6.0 × 6.0 mm scans, exhibited insignificant signs. Outer Retina and Choriocapillaris (CC) revealed a regional dark area centrally, corresponding to flow signal voids in a typical moth-eaten appearance, signifying areas of choroidal non-perfusion (Fig. 1C). In addition, a few hyperreflective lesions surrounding the focal dark area in the CC slab were observed (Fig. 1C).

Blood work up including complete blood count, erythrocyte sedimentation rate, chest radiography, serum ACE, lysozyme and Quantiferon, was normal. In addition, other bacterial (syphilis IgG, cat-scratch disease,) viral (HSV, VZV, CMV) and parasitic (toxoplasmosis, toxocariasis) infections were tested negative during blood exams. Based on the aforementioned findings along with patient’s elevated blood pressure the diagnosis of hypertensive choroidopathy due to malignant hypertension was made. Patient was referred to a general hospital for urgent medical care and was admitted to the hospital for 2 days. Blood pressure was controlled and secondary causes of malignant hypertension were excluded after a thorough examination in the cardiology department.

**Fig. 3:**
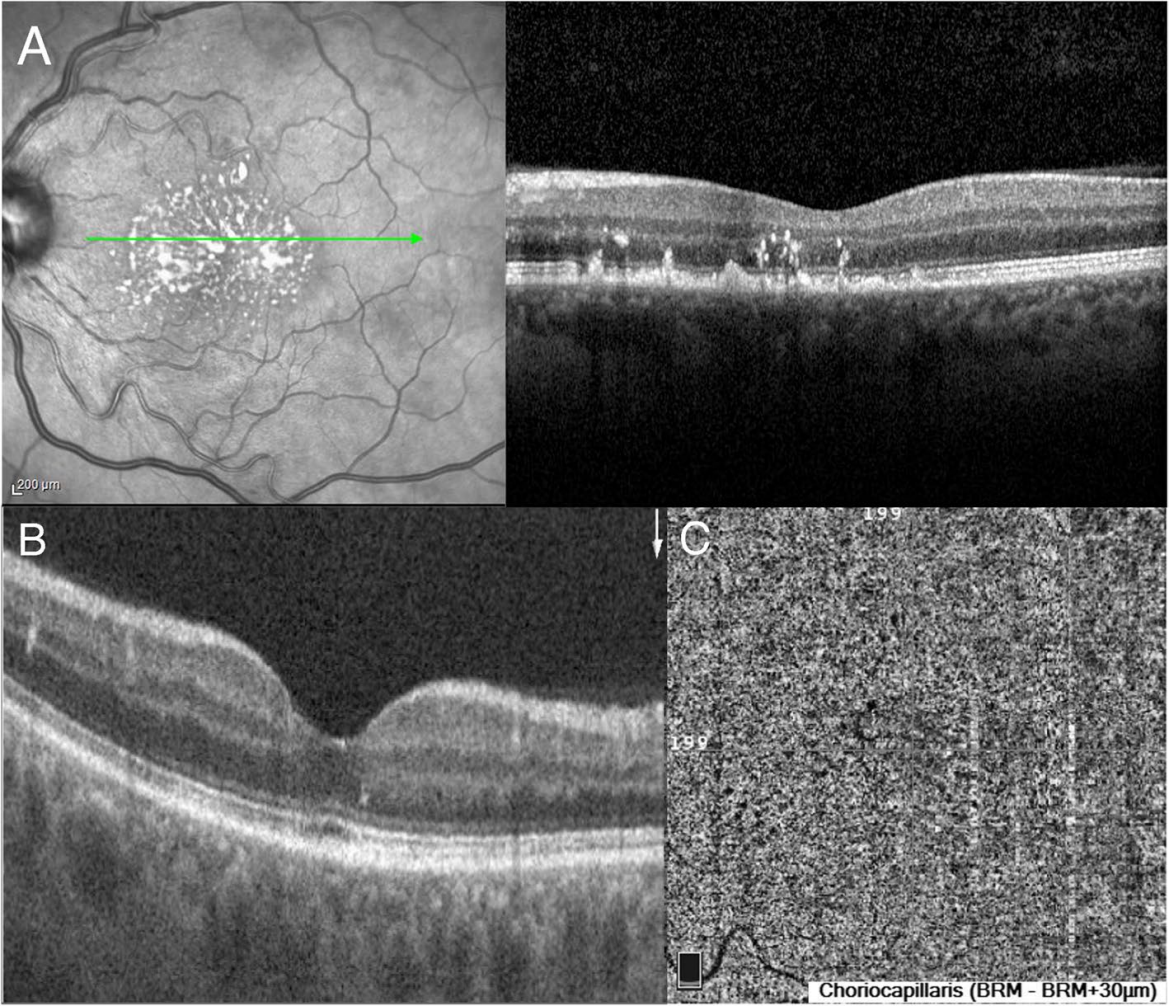
Exams at follow up **A**) SS- OCT scan at 2 months shows complete regression of exudative retinal detachment. Hard exudates aligned in a star like pattern and hyperreflective material overlying the RPE are also observed. **B** OCT scan at 9 months follow up exhibits no subretinal fluid and complete regression of hyperreflective material over the RPE. **C** OCTA shows utter restoration of the choriocapillaris with no flow voids. Vessel density returns to normal levels and no hyperreflective lesions are observed

During the 9 months follow-up period, patient's BP was controlled with antihypertensive treatment and measured 130/85 mmHg while visual acuity was 20/20 in both eyes. Fundoscopy showed hard exudates aligned in a star-like pattern, that were not present at baseline examination and complete resolution of the initial retinal detachment. SS-OCT B-scan demonstrated hard exudates and hyperreflective material overlying the Retinal Pigment Epithelium (RPE) that slowly disappeared during the 9-month period (Fig. 3A, B). CC slab in OCTA indicated normal perfusion of the choroidal vessels with no flow voids while vessel density had been completely restored compared to the initial exam (Fig. 3C).

## Discussion

Hypertensive emergencies can be manifested first in the eyes. Hypertensive chorioretinopathy is observed more commonly in younger patients with acute elevation of blood pressure and is usually associated with conditions such as malignant hypertension, pre-eclampsia, eclampsia, acute or chronic renal failure [2, 3, 5]. A delayed diagnosis could be crucial not only for patient’s visual prognosis but also for irreversible cardiovascular and renal damage. Patient in this case was older and mentioned no past medical conditions, complexing the differential diagnosis.

Differential diagnosis in such cases is always challenging. Diseases such as systemic lupus erythematosus choroidopathy and Vogt- Koyanagi -Harada disease were excluded due to unilateral presentation, the absence of any ocular inflammatory signs and normal blood test results. Presence of intraretinal fluid on OCT and hyperfluorescent dot spots all over the posterior pole may resemble chronic Central Serous Retinopathy. However, patient’s acute onset of symptoms as well as the reduced visual acuity lead to the diagnosis of acute choroidopathy which was confirmed by the measurement of BP.

Hypertensive choroidopathy is an unusual, individual finding in patients with malignant hypertension, who often present with retinal findings such as hemorrhages, cotton wool spots, hard exudates, microaneurysms and/or papilledema [3]. Christopher Seungkyu Lee et al.2019 retrospective data, suggest that hypertensive retinopathy features are present in 97,8% (44/45) of their subjects with malignant hypertension and are associated with poor visual outcomes [5]. These signs were not present in this particular case making the differential diagnosis even more challenging. Visual acuity restoration of our patient, after adjusting blood pressure to normal levels is compelling, signifying that detection of choroidal involvement before expanding to the retina, is vital.

Indocyanine green angiography has long been considered the gold standard for illustrating choroidal vasculature, however it was contraindicated due to patient’s iodine allergy. Instead, OCT-angiography was utilized, in order to portray the choroid. OCTA scans the eye and captures the choriocapillaris layer separately from other layers of the retina and the choroid. Only a few scientific reports have been involved with OCTA markers in hypertensive choroidopathy. Saito et al. 2017 [6], Rezkallah et al. 2019 [4] and Ben Mrad et al. 2019 [7] compare choroidal ischemic areas and reperfusion, before and after treatment in patients with hypertensive choroidopathy, using OCTA. CC slabs demonstrate dark areas with flow voids that are being gradually restored following treatment, after 1 month. The findings in this patient are in accordance with these studies. In addition, a 9 month follow up is presented, until utter regeneration of choroidal perfusion and complete restoration in vessel density is noticed.

Rotsos et al. 2019 [8], described 2 cases of hypertensive choroidopathy with multiple hyperreflective lesions visible both in structural OCT and OCTA CC slab at baseline and 1 month follow up. According to the researchers, these irregular shaped lesions correspond to fibrin deposits overlying the RPE. This finding was also observed in this patient at initial examination and 2-month visit, but was no longer present at the 9-month visit.

Focal ischemia of the RPE due to non-perfusion of the underlying choriocapillaris, is the main pathophysiological mechanism in hypertensive choroidopathy [3]. Exudative retinal detachment is the result of RPE pump malfunction and Elschnig spots are ischemic areas at the RPE level, that appear yellow with RPE hyperpigmentation and a surrounding halo of hypopigmentation [3]. Marta Ugarte et al. 2008 [2] and Rotsos et al. 2019 [8], report cases of hypertensive choroidopathy with exudative retinal detachments, multiple pigment epithelial detachments and Elschnig spots. Patients develop persistent visual loss, due to permanent damage of the RPE. According to experimental studies, RPE has an essential role in CC maintenance [9] and RPE degeneration can lead to choriocapillaris atrophy as reported by D. Scott McLeod et al. 2009 [10]. However, in this particular case there were no such signs of permanent RPE impairment, as Elschnig spots and Retinal Pigment Epithelial Detachments. During the follow up period, RPE remains intact as seen in structural OCT, thus the choriocapillaris manage to regenerate, as we clearly observe in the OCTA.

## Conclusion

Malignant hypertension is a medical emergency that can firstly affect the eye. Hypertensive choroidopathy with exudative retinal detachment may be the only manifestation of the disease and no pre-existing history of a systemic disease is required in order to become apparent. Thus, it would be wise for ophthalmologist to always check the blood pressure when patients present with unusual ophthalmic signs.

The findings of this study suggest that even though visual function recovery and anatomical restoration of the retina is remarkable during the two-month follow up, it takes a longer period for the choroid to fully heal. OCTA can be very practical in the diagnosis and follow-up of patients with hypertensive choroidopathy, especially when conventional angiographies are contraindicated. Further studies with multiple cases will illuminate the exact role of OCTA in choroidopathies.

## Data Availability

Data sharing is not applicable to this article as no data sets were generated or analysed during the current study.

## Abbreviations

BP: Blood Pressure
OCTA: Optical Coherence Tomography Angiography
SD-OCT: Spectral Domain Optical Coherence Tomography
SS-OCT: Swept Source Optical Coherence Tomography
FA: Fluorescein Angiography
CC: Choriocapillaris
RPE: Retinal Pigment Epithelium
